# Center-To-Periphery Arterial Stiffness Gradient Is Attenuated and/or Reversed in Pregnancy-Associated Hypertension

**DOI:** 10.3389/fcvm.2021.766723

**Published:** 2021-12-24

**Authors:** María M. Pereira, Juan Torrado, Claudio Sosa, Alejandro Diaz, Daniel Bia, Yanina Zócalo

**Affiliations:** ^1^Department of Obstetrics and Gynecology, BronxCare Hospital Center a Clinical Affiliate of Mt Sinai Health Systems and Academic Affiliate of Icahn School of Medicine, Bronx, NY, United States; ^2^Department of Internal Medicine, Jacobi Medical Center, Albert Einstein College of Medicine, Bronx, NY, United States; ^3^Department of Obstetrics and Gynecology “C”, Pereira-Rossell Hospital, School of Medicine, Republic University, Montevideo, Uruguay; ^4^Consejo Nacional de Investigaciones Científicas y Técnicas (CONICET), Tandil, Argentina; ^5^Department of Physiology, Centro Universitario de Investigación, Innovación y Diagnóstico Arterial (CUiiDARTE), School of Medicine, Republic University, Montevideo, Uruguay

**Keywords:** arterial stiffness, carotids, gestational hypertension, pregnancy, pre-eclampsia

## Abstract

**Background:** Non-pregnant (NP) women have a progressive increase in arterial stiffness from central-to-peripheral arteries [“stiffness gradient” (SG)], which is of physiologic importance since excessive pulsatility is filtered by the creation of wave reflections. If the aorta gets stiff with minimal or no change in the periphery, the SG is dissipated transmitting pressure disturbances to the microcirculation. It remains unknown the status of the SG in both women with healthy pregnancies (HP) and complicated by pregnancy-associated hypertension (PAH).

**Objective:** To determine whether HP and PAH are associated with changes in SG. Secondarily, we aim at identifying potential differences between the subgroups of PAH (pre-eclampsia and gestational hypertension).

**Methods:** HP (*n* = 10), PAH (*n* = 16), and healthy NP women (*n* = 401, to be matched for age, and cardiovascular risk with the pregnant women) were included. Carotid-to-femoral (cfPWV) and carotid-to-radial pulse wave velocity (crPWV), common carotid artery (CCA) and brachial artery (BA) diameters and elastic modulus (EM), and regional (cfPWV/crPWV or “PWV ratio”) and local (CCA EM/BA EM or “EM ratio”) SG were quantified.

**Results:** HP showed no changes in PWV ratio compared with NP, in the presence of significantly lower cfPWV and crPWV. HP exhibited higher arterial diameters and lower CCA EM/BA EM compared to NP, without differences with PAH. PAH was associated with a significant increase in the PWV ratio that exceeded the levels of both NP and HP, explained by a lower (although significant) reduction of cfPWV with respect to that observed in HP with respect to NP, and a higher reduction in crPWV with respect to that observed between HP and NP. The blunted reduction in cfPWV observed in PAH coincided with an increase in the CCA EM.

**Conclusions:** Compared with NP, HP was associated with unchanged PWV ratio but with a reduction in CCA EM/BA EM, in the setting of a generalized drop in arterial stiffness. Compared with NP and HP, PAH was associated with an “exaggerated rise” in the PWV ratio without changes in CCA EM/BA EM, in the setting of a blunt reduction in cfPWV but exaggerated crPWV drop. The SG attenuation/reversal in PAH was mainly driven by pre-eclampsia.

## Introduction

Pre-eclampsia (PE) and gestational hypertension (GH), collectively denominated as pregnancy-associated hypertension (PAH), complicate 3–8% of all pregnancies with serious short- and long-term maternal and neonatal consequences (1–3). While some evidence suggests that GH and PE may represent different disorders, several investigations support that GH could in fact be a milder manifestation of PE, and that these conditions are a continuous spectrum of the same pregnancy-induced syndrome (1, 3). Yet, the pathophysiology of PAH remains to be fully elucidated. The most accepted theory proposes that an impaired placentation (i.e., shallow invasion of trophoblast of the spiral arteries) results in a dramatic rise in the resistance of the uterine-placental vasculature, which leads to a rapid development of a disproportionate rise in blood pressure (BP), inappropriate inflammatory response, generalized endothelial dysfunction, and multi-organ damage (“placental origin hypothesis”) (4). However, this hypothesis was recently questioned, since placental histopathology lesions thought to be characteristic of this condition are neither sensitive nor specific markers for the disorder (5, 6).

Recent evidence has revealed that PAH may develop in those women that are not able to develop an optimal cardiovascular adaptation to the naturally occurring hemodynamic loads of pregnancy (“cardiovascular origin hypothesis”) (5, 7). In this hemodynamic context (e.g., increased BP and arterial stiffness), excessive pulsatility in the central arteries may be preferentially transmitted to the placental microvasculature (“secondary placental dysfunction”), as well as to other vulnerable circulations. Recently, our group reviewed the available evidence and concluded that both theories (“placental and cardiovascular origin”) may be complementary and, a combined theory may be reasonable for most women (5). However, although the analysis of the current literature supports that PAH would be associated with several arterial abnormalities that would prevent an optimal reduction of the peripheral propagation of the pressure pulsatility, being this a potential link between both theories, many of these pathophysiologic clues remain to be confirmed through studies that simultaneously assess different regions of the vascular system.

Since healthy pregnancy (HP) is characterized by a pronounced increase in stroke volume (SV) and cardiac output (CO) to meet metabolic requirements, low aortic stiffness plays a key role in preventing large increases in pulse pressure (PP) and systolic BP (SBP) during pregnancy (8). In normal conditions, forward pressure waves generated intermittently by the left ventricle (LV) and re-reflections of backward propagating waves at the ventricular-aorta interface, travel throughout the arterial system and collide with reflection sites located in the arterial tree and reflect to the center as backward or retrograde pressure waves (9, 10). Pressure wave reflections can originate in multiple sites, such as arterial narrowing and bifurcations, but particularly, where the arterial stiffness (AS) of one region changes to another [“stiffness gradient” (SG)] (8–10). In healthy non-pregnant (NP) populations, there is a progressive increase in the AS from the aorta to the peripheral arteries, which is of physiologic importance since creates partial wave reflections reducing the energy carried peripherally by the forward wave to the microcirculation (11, 12). If the aorta becomes stiff with minimal or no changes in the peripheral vessels, the SG is dissipated (or reversed) increasing the risk of transmitting high pulsatile energy preferentially to low-resistance vascular beds (13–15). Aortic stiffness was found consistently elevated in PAH compared to HP in several cross-sectional (16–19) and prospective studies (20, 21), whereas changes in peripheral vascular stiffness have been a matter of debate. Yet, it remains unknown the status of the SG in women with PAH, which would have a paramount role in decreasing distal pulsatility and protecting the microcirculatory beds.

**Figure 1:**
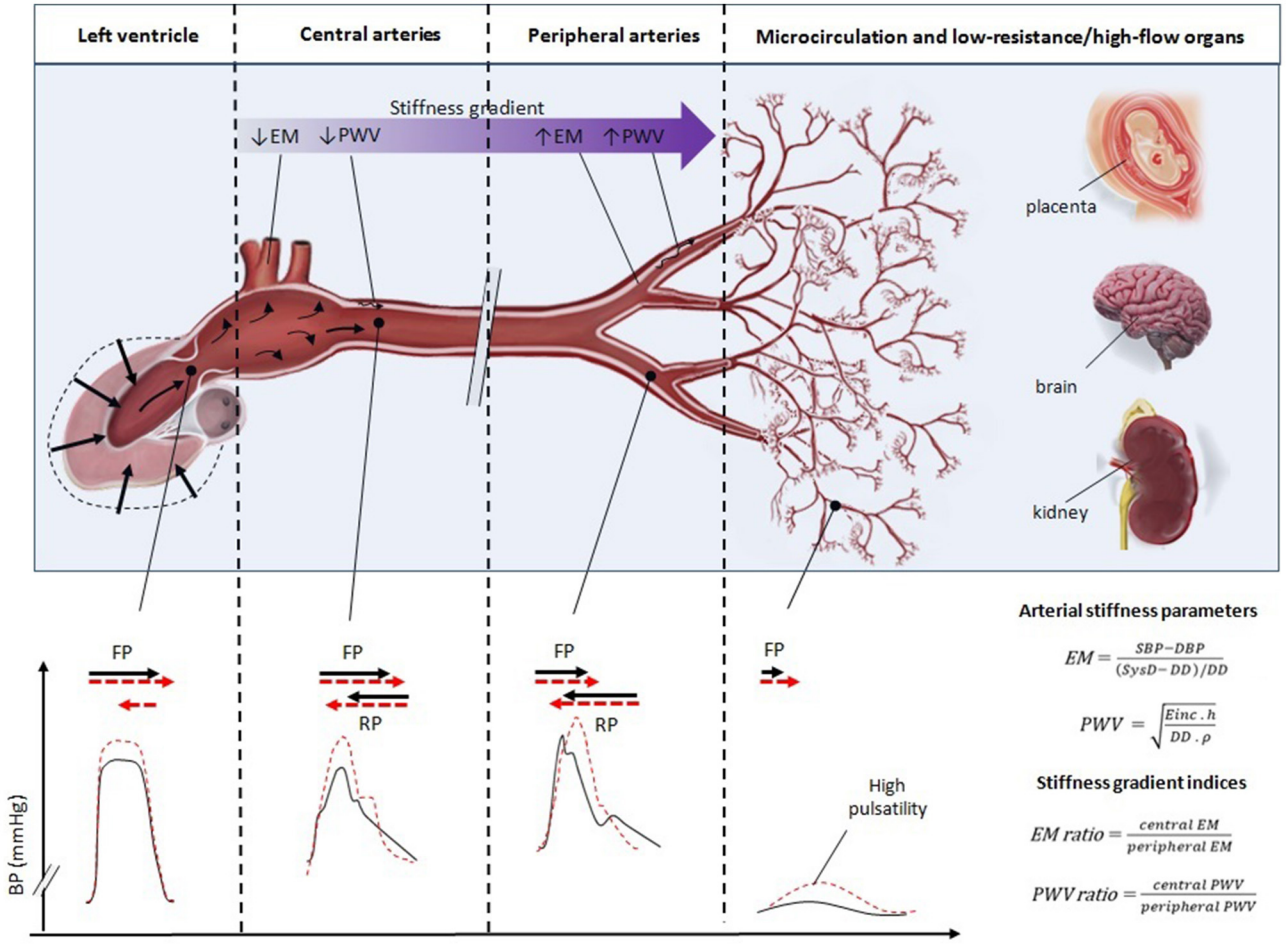
Schematic representation of the interplay between central (e.g., aortic or carotid) and peripheral (e.g., brachial) arterial stiffness in determining the arterial stiffness gradient, partial wave reflection, and distal microcirculatory pressure pulsatility. Normally, the arterial system is characterized by a progressive increase in the arterial stiffness from the aorta to the peripheral arteries (“stiffness gradient”), which can be estimated by PWV (Moens–Korteweg equation), EM and their respective ratios (note that these parameters are inversely determined by the arterial diameter). This gradient creates partial wave reflection [i.e., retrograde pressure waves (RP)] reducing the energy carried peripherally by the forward pressure wave (FP) protecting the microcirculation. With aortic stiffening and/or peripheral maladaptation, the stiffness gradient dissipates (represented by progressive higher levels of PWV and/or EM), the aorta does not optimally distend increasing left ventricle (LV) afterload [represented by increased LV and aortic pressure (dashed red lines)], and increasing the likelihood of high pulsatile energy transmission (dashed red lines) to low-resistance/high-flow vascular beds, such as renal, cerebral, or placental circulation.

Since the speed of pressure wave propagation in a specific arterial region is proportional to its stiffness, the measurement of pulse wave velocity (PWV) has been conveniently used as a surrogate of regional AS (Figure 1) (8–10). Moreover, the PWV ratio [i.e., carotid-to-femoral PWV (cfPWV)/carotid-to-radial PWV (crPWV)], the relationship between the stiffness of central elastic and muscular peripheral arterial pathways, has been used as a trustworthy tool to evaluate the SG, being independently associated with increased mortality in kidney disease, stroke, and hypertension (11–13, 22). Alternatively, the SG could be also quantified by using local measures of AS [e.g., elastic modulus (EM)]. In theory, local indices of AS could be more sensitive to detect incipient (mild) vascular changes compared with regional indices (i.e., PWV ratio). Moreover, since PWV and EM are inversely determined by increases in arterial diameters (the larger the diastolic diameter the lower the PWV, but the higher EM) (Figure 1), which characteristically occur during pregnancy, local parameters could also offer complementary information in these patients.

This study sought to determine whether HP and PAH are associated with different changes in the physiological center-to-periphery SG assessed by regional (PWV ratio) and local parameters (EM ratio) compared with an NP population. Secondarily, we identified potential differences between the subgroups of PAH (i.e., PE and GH).

## Materials and Methods

### Subjects

This was a cross-sectional study involving non-pregnant and pregnant women from our CUiiDARTE Project database (23–32). The CUiiDARTE Project is a population-based study developed in Uruguay, supported by the National Public Health Ministry and the Agencia Nacional de Investigación e Innovación (ANII). Cardiovascular evaluation in the CUiiDARTE Project involves a stepwise protocol using several equipment and devices that measure central, peripheral, and systemic hemodynamic variables, and structural and functional local, regional, and global properties of elastic, muscular, and transitional arteries (23–32). All procedures and protocols were conducted in agreement with the Declaration of Helsinki, approved by the Institution's Ethics Committee. Written informed consent was obtained prior to the examination.

Healthy NPs (*n* = 401) were selected to be matched for age and global cardiovascular risk factors (CRFs) with the below-mentioned pregnant women. By using propensity score matching methods, efficient matching and balance are created on the mentioned covariates and their confounding effect can be minimized or entirely removed (13). HP women (*n* = 10), without known family history of premature cardiovascular disease (CVD), were recruited from the routine antenatal clinic. All women had uncomplicated pregnancies before and during the study. Women with PAH (*n* = 16) were recruited from the antenatal hospital ward, where they were admitted due to mild hypertension [brachial artery BP (baBP) 140/90–149/109 mmHg].

According to the Bulletin of the American College of Obstetricians and Gynecologists (1), PAH is defined as baBP >140/90 mmHg on two consecutive occasions more than 4 h apart, developing after 20 weeks of gestation in a previously normotensive woman. Depending on whether there was or not significant proteinuria (≥300 mg per 24-h urine collection), patients were further classified as PE or GH, respectively, since all patients included in the study had no evidence of severe features. Laboratory samples were obtained prior to the study enrollment. A clinical interview, together with the anthropometric evaluation [body weight (BW), body height (BH), and body mass index (BMI)] enabled us to assess CRF exposure. A family history of CVD was defined by the presence of first-degree relatives with early CVD (<55 years in men; <65 years in women). Women were categorized as “sedentary” [according to the WHO physical activity (PA) recommendations] if they performed <150 min of moderate-intensity aerobic PA, or <75 min of vigorous-intensity aerobic PA, or an equivalent combination of moderate-intensity and vigorous-intensity PA throughout the week. Dyslipidemia was defined as self-reported or total cholesterol ≥240 mg/dL or HDL cholesterol <46 mg/dL (if available).

### Non-Invasive Arterial Evaluation

Subjects were instructed to lie in a left lateral position (to avoid the compression of the inferior vena cava in the case of pregnant women) in a temperature-controlled (21–23°C) room, for at least 10–15 min, to establish a stable hemodynamic condition. Once heart rate (HR) and baBP were stabilized, non-invasive arterial parameters were obtained as described below.

#### Carotid-to-Femoral PWV, Carotid-to-Radial PWV, and PWV Ratio

Carotid-to-femoral pulse wave velocity and crPWV were non-invasively measured to assess the aortic and upper-limb regional stiffness (SphygmoCor 7.01, AtCor Medical, Sydney, Australia). PWV ratio (regional SG) was calculated as: cfPWV/crPWV (13, 23).

#### Local Arterial Stiffness and Ratio

Left and right common carotid artery (CCA) and left brachial artery (BA) were analyzed using high-resolution ultrasound (6–13 MHz, MicroMaxx/M-Turbo, Sonosite Inc., WA, USA). CCA and BA blood flow velocity levels and indexes were quantified (26). Sequences of images (30 s, B-Mode, longitudinal views) were processed to obtain peak systolic and end-diastolic diameters (SysD, DD) and CCA intima–media thickness (IMT; far wall, end-diastole) (32). CCA diameters and IMT were measured a centimeter proximal to the bulb, while BA measurements were obtained at the elbow level in a straight segment of at least 1 cm (32). Carotid arteries were also screened for the presence of atheroma plaques (23, 25, 26, 31). Local stiffness was quantified by EM (32):


$$EM=\frac{\left(SBP-DBP\right)}{(SysD-DD)/DD,},$$

where SBP and DBP are systolic and diastolic BP. The EM measures the ability of the arteries to change their dimensions in response to the PP caused by the cardiac ejection [pressure change required for (theoretic) 100% increase in diameter]. Oscillometry-derived baSBP and baDBP (HEM-433INT Oscillometric System; Omron-Healthcare Inc., Illinois, USA) were used to quantify BA EM, whereas aortic SBP and DBP (aoSBP, aoDBP) were used to quantify CCA EM. Radial pulse waves were obtained by the applanation tonometry (SphygmoCor 7.01, AtCor-Medical, Sydney, Australia), and calibrated to baDBP and mean BP [MBP = baDBP + (baSBP–baDBP)/3]. The aoSBP and aoDBP were derived from the radial recordings using a general transfer function (30, 32). Finally, local SG was calculated as the quotient between left and right CCA EM (and its average) and BA EM (CCA EM/BA EM).

### Data Analysis

Normality of the distribution of the data was examined using the Shapiro–Wilk test and Q–Q plot. The *p* < 0.05 indicates significant statistical differences. One-way analysis of covariance (ANCOVA) with multiple adjusted comparisons was utilized for the evaluation of differences in cardiovascular variables. Demographic characteristics (age), anthropometric (BW, BH, BMI), CRFs exposure, and medication use of the participants were considered as adjustment variables. Considering the relatively small sample sizes of the HP and PAH groups, we performed bootstrapping of the samples, as a strategy to evaluate whether potential statistical differences observed between the study groups maintain even after analyzing different random sampling settings. To this end, bootstrap-derived 95% CIs (1,000 samples) were obtained applying bias-corrected and accelerated methods for computing CI limits (32). In other words, with this mechanism, any initial *p* < 0.05 may no longer be significant after the “fictional random re-sampling” (i.e., bootstrapping). This type of test obligates the investigators to consider only those significant *p*-values that replicate in both statistical scenarios (i.e., the actual sample and bootstrapping sampling). After performing the comparative analyses between the three primary groups (NP, HP, and PAH), as secondary analysis, we further investigate differences between the four groups (NP, HP, GH, and PE) by discriminating women with PE and GH within the PAH group.

The statistical analyses were performed using the Statistical Package for Social Sciences (version 26.0). All data were presented as mean value (MV) ± standard deviation (SD) or error (SE) of the mean, as well as lower and upper limits (LL and UL, respectively).

## Results

### Regional and Local Arterial Stiffness Gradient of NP, HP, and PAH

Descriptive characteristics and baseline cardiovascular parameters of the study groups are presented in Table 1 and Supplementary Table 1. No women had carotid plaques, diabetes, or family history of premature CVD. Regardless of age and classic CRFs exposure, HP was associated with non-significant changes in PWV ratio compared with NP (assessed by bootstrapping), but with lower levels compared with PAH (Table 2). This finding was observed in the presence of significantly lower levels of cfPWV compared with both NP and PAH. HP was also associated with lower levels of crPWV compared with NP, but with only a trend of showing lower crPWV values compared with PAH (*p*: 0.06–0.08; Table 2).

**Table 1:** Demographic, anthropometric, clinical, and cardiovascular characteristics according to the study groups.

	**Non-Pregnant women**					**Healthy pregnant women**					**Pregnancy-Associated hypertension**				
**Variables**	**MV**	**SD**	**SE**	**Min**	**Max**	**MV**	**SD**	**SE**	**Min**	**Max**	**MV**	**SD**	**SE**	**Min**	**Max**
Age (years)	22.84	5.99	0.30	18	40.7	29.40	6.17	1.95	21.00	40	32.3	6.2	1.6	20.0	40.0
Body weight (kg)	60.75	12.05	0.62	38.10	128.00	67.10	7.32	2.32	56.00	77.00	86.75	15.53	3.88	60.00	113.00
Body height (m)	162.22	6.38	3.25	132.00	183.00	157.60	6.75	2.14	148.00	173.00	159.94	6.93	1.73	149.00	174.00
BMI (kg/m^2^)	23.04	4.23	0.22	16.50	48.77	27.13	3.56	1.13	20.72	31.23	34.16	7.12	1.78	21.77	48.27
Hypertension (%)	4.0					0					100				
On BP treatment (%)	1.7					0					12.5				
Dyslipidemia (%)	7.2					20.0					0				
Sedentarism (%)	58.6					100					100				
**Peripheral and central hemodynamic parameters**															
baSBP (mmHg)	118	11	1	85	177	114	5	2	106	121	127	12	3	98	143
baDBP (mmHg)	69	9	1	49	103	65	10	3	52	85	75	9	2	59	93
Heart rate (bpm)	75	11	1	50	113	81	16	5	52	97	87	13	3	66	110
aoSBP (mmHg)	102	10	1	79	156	100	7	2	90	109	110	11	3	84	126
aoDBP (mmHg)	70	9	1	51	103	67	10	3	55	87	77	9	2	60	94
**Carotid and brachial structural and hemodynamic parameters**															
Left CCA SysD (mm)	6.61	0.49	0.03	4.84	9.55	7.08	0.39	0.12	6.13	7.49	7.13	0.50	0.12	5.78	7.98
Left CCA DD (mm)	6.04	0.47	0.03	4.39	8.40	6.47	0.42	0.13	5.75	7.19	6.64	0.47	0.12	5.55	7.54
Left CCA IMT (mm)	0.504	0.090	0.005	0.311	1.352	0.527	0.082	0.026	0.422	0.670	0.564	0.095	0.024	0.387	0.774
Left CCA PSV (cm/s)	97.42	19.31	1.09	55.80	191.90	85.15	21.11	10.56	68.50	116.10	93.95	22.88	7.23	59.00	144.60
Left CCA EDV (cm/s)	26.72	5.99	0.34	5.41	49.70	29.35	5.34	2.67	22.20	34.30	25.77	3.65	1.15	20.60	31.70
Left CCA RI	0.72	0.06	0.00	0.55	0.93	0.65	0.09	0.04	0.56	0.72	0.72	0.06	0.02	0.62	0.82
Left CCA SDR	3.78	1.03	0.06	2.20	13.66	2.94	0.70	0.35	2.27	3.58	3.65	0.77	0.24	2.64	5.44
Right CCA SysD (mm)	6.71	0.52	0.03	5.20	9.52	7.10	0.60	0.19	6.07	7.86	7.08	0.57	0.14	5.88	7.90
Right CCA DD (mm)	6.15	0.49	0.03	4.53	8.48	6.49	0.59	0.19	5.58	7.22	6.58	0.52	0.13	5.51	7.49
Right CCA IMT (mm)	0.502	0.084	0.005	0.304	1.218	0.480	0.089	0.028	0.365	0.605	0.597	0.174	0.043	0.335	1.159
Right CCA PSV (cm/s)	93.58	18.91	1.08	50.50	161.60	96.55	38.09	19.05	66.60	152.20	84.86	23.16	6.98	32.70	127.50
Right CCA EDV (cm/s)	25.91	6.47	0.37	9.01	56.60	26.60	2.69	1.34	24.70	30.40	23.45	4.68	1.41	13.70	28.50
Right CCA RI	0.72	0.06	0.00	0.53	0.88	0.70	0.08	0.04	0.60	0.80	0.71	0.07	0.02	0.58	0.79
Right CCA SDR	3.73	0.90	0.05	2.12	8.44	3.57	1.05	0.53	2.50	5.01	3.61	0.73	0.22	2.39	4.79
Left BA SysD (mm)	3.38	0.43	0.06	2.27	4.63	3.80	0.32	0.10	3.11	4.17	4.10	0.47	0.12	3.26	4.72
Left BA DD (mm)	3.21	0.44	0.06	2.02	4.44	3.65	0.34	0.11	2.90	4.05	3.90	0.46	0.11	3.09	4.52
Left BA PSV (cm/s)	75.75	16.89	2.00	37.30	127.10	58.80	16.40	11.60	47.20	70.40	115.99	24.12	9.12	83.70	146.50
Left BA EDV (cm/s)	−2.19	10.97	1.30	−35.30	20.30	15.02	8.32	5.89	9.13	20.90	23.30	6.41	2.42	15.20	34.30
Left BA RI	0.99	0.08	0.01	0.80	1.30	0.76	0.08	0.06	0.70	0.81	0.80	0.04	0.01	0.73	0.84
**Arterial stiffness and stiffness gradient parameters**															
cfPWV (m/s)	6.48	1.02	0.06	4.10	10.52	5.49	0.69	0.22	4.11	6.58	6.55	0.96	0.24	5.00	8.96
crPWV (m/s)	8.83	1.40	0.12	5.10	12.90	7.18	1.55	0.49	5.24	9.64	6.38	1.07	0.27	4.69	8.21
PWV Ratio	0.71	0.11	0.01	0.49	1.03	0.79	0.17	0.05	0.56	1.05	1.06	0.25	0.06	0.78	1.50
Left BA EM (mmHg)	1,176	729	100	267	3,921	1,445	592	187	591	2,393	1,158	455	114	430	1,978
Left CCA EM (mmHg)	357	116	6	173	831	361	129	43	237	647	502	183	46	246	790
Right CCA EM (mmHg)	364	116	7	173	913	345	69	23	240	438	492	195	49	295	878
Mean CCA EM (mmHg)	360	107	6	188	747	353	93	31	239	529	497	183	46	290	834
Left CCA EM/BA EM	0.43	0.29	0.04	0.06	1.68	0.28	0.17	0.006	0.14	0.63	0.51	0.27	0.07	0.17	1.04
Right CCA EM/BA EM	0.45	0.28	0.04	0.09	1.33	0.26	0.11	0.04	0.14	0.41	0.49	0.26	0.07	0.15	1.07
Mean CCA EM/BA EM	0.44	0.27	0.04	0.09	1.37	0.27	0.14	0.05	0.15	0.51	0.50	0.26	0.07	0.17	1.02

*MV, mean value; SD, standard deviation; SE, standard error; Min, minimal value; Max, maximal value; BMI, body mass index; SBP, DBP, systolic and diastolic blood pressure, respectively; ao, aortic; ba, brachial artery; CCA, common carotid artery; BA, brachial artery; SysD, DD, peak systolic and end-diastolic diameter, respectively; IMT, intima-media thickness; PSV, EDV, peak systolic- and end-diastolic velocity, respectively; RI, resistive index; SDR, systo-diastolic ratio; cfPWV, crPWV, carotid-to-femoral and carotid-to-radial pulse wave velocity; PWV, pulse wave velocity; EM, elastic modulus; Sample size, Non-pregnant women (n = 401), Healthy pregnant women (n = 10), Pregnancy-associated hypertension (n = 16)*.

**Table 2:** Regional and local arterial stiffness and gradient: comparison after adjustments (ANCOVA).

	**After Adjustment**					**Pair wise Comparisons**			
**Variable**	**Group**	**MV**	**SE**	**LL**	**UL**		**NP vs. HP**	**NP vs. PAH**	**HP vs. PAH**
**Regional arterial stiffness and gradient**									
PWV ratio	NP	0.71	0.01	0.69	0.74	*p*	0.046	<0.001	<0.001
	HP	0.80	0.05	0.71	0.89	Boot. *P*	0.060	<0.001	0.003
	PAH	1.05	0.04	0.97	1.13		–	–	–
cfPWV (m/s)	NP	6.51	0.05	6.41	6.61	*p*	<0.001	0.017	0.014
	HP	5.16	0.29	4.59	5.74	Boot. *P*	<0.001	0.025	0.004
	PAH	5.99	0.24	5.52	6.46		–	–	–
crPWV (m/s)	NP	8.95	0.12	8.72	9.18	*P*	<0.001	<0.001	0.077
	HP	6.59	0.45	5.69	7.48	Boot. *P*	<0.001	<0.001	0.066
	PAH	5.77	0.39	5.00	6.53		–	–	–
**Local arterial stiffness and gradient**									
Left BA DD (mm)	NP	3.20	0.06	3.08	3.33	*P*	0.003	<0.001	0.178
	HP	3.68	0.16	3.37	3.99	Boot. *P*	0.002	<0.001	0.165
	PAH	3.87	0.12	3.63	4.11		–	–	–
Right CCA DD (mm)	NP	6.14	0.03	6.09	6.20	*P*	0.051	0.003	0.308
	HP	6.40	0.16	6.10	6.71	Boot. *P*	0.099	0.005	0.344
	PAH	6.50	0.13	6.25	6.75		–	–	–
Left CCA DD (mm)	NP	6.04	0.03	5.99	6.09	*p*	0.015	<0.001	0.218
	HP	6.37	0.15	6.08	6.67	Boot. *P*	0.009	<0.001	0.211
	PAH	6.52	0.12	6.28	6.76		–	–	–
Mean CCA EM/BA EM	NP	0.46	0.04	0.39	0.53	*p*	0.071	0.415	0.147
	HP	0.31	0.09	0.13	0.49	Boot. *P*	0.046	0.400	0.092
	PAH	0.44	0.07	0.30	0.57		–	–	–
Left CCA EM/BA EM	NP	0.45	0.04	0.38	0.53	*P*	0.123	0.460	0.192
	HP	0.33	0.10	0.14	0.52	Boot. *P*	0.097	0.459	0.144
	PAH	0.44	0.07	0.30	0.59		–	–	–
Right CCA EM/BA EM	NP	0.46	0.04	0.39	0.53	*p*	0.050	0.379	0.129
	HP	0.29	0.09	0.11	0.48	Boot. *P*	0.041	0.372	0.106
	PAH	0.44	0.07	0.30	0.57		–	–	–
Left CCA EM (mmHg)	NP	360.51	5.95	348.80	372.23	*p*	0.156	0.023	0.010
	HP	308.81	35.83	238.32	379.30	Boot. *P*	0.117	0.122	0.033
	PAH	425.71	27.74	371.15	480.28		–	–	–
Right CCA EM (mmHg)	NP	368.18	5.91	356.54	379.81	*p*	0.078	0.011	0.005
	HP	282.65	35.36	213.09	352.20	Boot. *P*	0.058	0.061	0.016
	PAH	416.39	27.33	362.63	470.15		–	–	–
Left BA EM (mmHg)	NP	1,158.85	92.37	974.67	1,343.03	*P*	0.374	0.218	0.400
	HP	1,239.06	230.37	779.71	1,698.40	Boot. *P*	0.366	0.133	0.381
	PAH	1,318.20	178.89	961.50	1,674.90		–	–	–

*MV, mean value; SE, standard error; 95% CI, 95% confidence interval; LL, lower limit; UL, upper limit; Boot, bootstrapping; NP, non-pregnant women (n = 401); HP, healthy pregnant women (n = 10); PAH, pregnancy-associated hypertension (n = 16); CCA, common carotid artery; BA, brachial artery; DD, end-diastolic diameter; cfPWV, crPWV, carotid-to-femoral and carotid-to-radial pulse wave velocity; PWV, Pulse wave velocity; EM, elastic modulus*.

When considering EM ratio, a general trend of lower values was observed in HP when compared with NP women, although no clear differences were observed when comparing with PAH (Table 2). HP showed significantly lower levels of CCA EM compared with PAH, without clear differences when compared with NP. No differences were observed in the BA EM compared with NP or PAH. Finally, HP showed higher levels of BA and CCA diameters than NP and was similar to PAH (Table 2). In summary, when comparing with NP status, HP was associated with a drop in the AS in both territories, evidenced by both regional (cfPWV and crPWV) and local (except for BA EM) stiffness-related parameters, without significant changes in PWV ratio (i.e., bootstrapping) but a reduction in CCA EM/BA EM.

PAH was associated with an increased in the PWV ratio that exceeded the levels of both NP and HP (Table 2). The rise in PWV ratio was explained by a lower (although significant) reduction of cfPWV levels with respect to that observed in HP with respect to NP, and a higher reduction in the levels of crPWV with respect to those observed between HP and NP. The greater drop in crPWV levels was followed by a trend toward higher levels in BA diameters (although not significant with respect to HP). The observed blunted reduction in cfPWV values in women with PAH coincided with an increase in the CCA EM compared with NP and HP (Table 2).

### Subgroup Analysis of PAH (Gestational Hypertension and Pre-Eclampsia)

When performing a subgroup analysis of women with PAH (Supplementary Table 1), the elevated stiffness ratio in PAH was mainly driven by the changes in arterial stiffness observed in those women with PE (Table 3). Indeed, although higher PWV ratio values were observed in GH compared to HP, these findings did not show statistical significance (*p* = 0.069 and *p* = 0.064; Table 3). However, women with PE were associated with exaggerated increase in PWV ratio compared with all other groups. PE showed higher and lower levels of cfPWV and crPWV, respectively, compared to GH (*p* < 0.05). Moreover, crPWV was found significantly reduced in women with PE compared with the other groups, where NP, HP, GH, and PE showed a descending order of crPWV values (Table 3). Women complicated with PE demonstrated higher levels of cfPWV compared with HP and GH, but similar to NP. Of note, the GH group showed a trend toward higher levels of PWV ratio and cfPWV compared with HP (*p*-values: 0.05–0.07; Table 3). Otherwise, GH presented similar values in almost all other analyzed parameters when compared to HP, while having similar differences with respect to NP.

**Table 3:** Regional and local arterial stiffness and gradient: comparison after adjustments (ANCOVA: 4 groups).

**Variable**	**Group**	**MV**	**SE**	**LL**	**UL**		**NP vs. HP**	**NP vs. GH**	**NP vs. PE**	**HP vs. GH**	**HP vs. PE**	**GH vs. PE**
**Regional arterial stiffness and gradient**												
PWV ratio	NP	0.71	0.01	0.69	0.74	*P*	0.027	<0.001	<0.001	0.069	<0.001	<0.001
	HP	0.80	0.04	0.72	0.89	Boot. *P*	0.044	<0.001	0.001	0.064	0.001	0.002
	GH	0.90	0.05	0.80	0.99		–	–	–	–	–	–
	PE	1.22	0.05	1.12	1.32		–	–	–	–	–	–
cfPWV (m/s)	NP	6.51	0.05	6.41	6.61	*P*	<0.001	0.002	0.402	0.177	0.002	0.028
	HP	5.17	0.29	4.59	5.74	Boot. *P*	<0.001	<0.001	0.418	0.059	0.001	0.019
	GH	5.57	0.32	4.93	6.21		–	–	–	–	–	–
	PE	6.42	0.33	5.78	7.07		–	–	–	–	–	–
crPWV (m/s)	NP	8.95	0.12	8.72	9.18	*P*	<0.001	<0.001	<0.001	0.321	0.022	0.059
	HP	6.57	0.45	5.68	7.46	Boot. *P*	<0.001	<0.001	<0.001	0.301	0.018	0.010
	GH	6.27	0.50	5.28	7.25		–	–	–	–	–	–
	PE	5.22	0.52	4.20	6.25		–	–	–	–	–	–
Local arterial stiffness and gradient												
Left BA DD (mm)	NP	3.20	0.06	3.08	3.33	*P*	0.003	0.002	<0.001	0.415	0.072	0.087
	HP	3.68	0.15	3.37	3.98	Boot. *P*	0.001	0.003	0.001	0.417	0.061	0.093
	GH	3.73	0.16	3.41	4.05		–	–	–	–	–	–
	PE	4.03	0.17	3.70	4.36		–	–	–	–	–	–
Right CCA DD (mm)	NP	6.14	0.03	6.09	6.20	*P*	0.051	0.054	0.007	0.461	0.223	0.259
	HP	6.40	0.16	6.10	6.71	Boot. *P*	0.087	0.076	0.009	0.468	0.247	0.258
	GH	6.43	0.17	6.09	6.77		–	–	–	–	–	–
	PE	6.58	0.18	6.24	6.93		–	–	–	–	–	–
Left CCA DD (mm)	NP	6.04	0.03	5.99	6.09	*P*	0.015	0.003	0.002	0.271	0.234	0.454
	HP	6.37	0.15	6.08	6.67	Boot. *P*	0.015	<0.001	0.013	0.204	0.258	0.454
	GH	6.51	0.17	6.18	6.84		–	–	–	–	–	–
	PE	6.54	0.17	6.20	6.87		–	–	–	–	–	–
Mean CCA EM/BA EM	NP	0.46	0.04	0.39	0.53	*P*	0.070	0.214	0.313	0.309	0.080	0.155
	HP	0.31	0.09	0.13	0.49	Boot. *P*	0.045	0.119	0.312	0.251	0.066	0.129
	GH	0.38	0.09	0.20	0.56		–	–	–	–	–	–
	PE	0.50	0.09	0.32	0.69		–	–	–	–	–	–
Left CCA EM/BA EM	NP	0.45	0.04	0.38	0.53	*P*	0.123	0.281	0.329	0.337	0.126	0.208
	HP	0.33	0.10	0.13	0.52	Boot. *P*	0.100	0.208	0.346	0.294	0.113	0.185
	GH	0.39	0.10	0.20	0.58		–	–	–	–	–	–
	PE	0.50	0.10	0.30	0.70		–	–	–	–	–	–
Right CCA EM/BA EM	NP	0.46	0.04	0.39	0.53	*P*	0.049	0.176	0.314	0.300	0.064	0.131
	HP	0.29	0.09	0.10	0.48	Boot. *P*	0.039	0.081	0.311	0.237	0.059	0.097
	GH	0.37	0.09	0.18	0.55		–	–	–	–	–	–
	PE	0.51	0.10	0.32	0.70		–	–	–	–	–	–
Left CCA EM (mmHg)	NP	360.43	5.88	348.86	372.01	*P*	0.079	0.382	<0.001	0.220	<0.001	0.001
	HP	309.43	35.41	239.77	379.09	Boot. *P*	0.055	0.399	0.005	0.222	0.002	0.017
	GH	349.04	37.46	275.36	422.72		–	–	–	–	–	–
	PE	504.85	38.02	430.06	579.64		–	–	–	–	–	–
Right CCA EM (mmHg)	NP	368.08	5.79	356.68	379.47	*P*	0.008	0.101	<0.001	0.231	<0.001	<0.001
	HP	283.40	34.64	215.25	351.54	Boot. *P*	<0.001	0.100	0.015	0.193	0.001	0.006
	GH	320.46	36.62	248.42	392.50		–	–	–	–	–	–
	PE	515.32	37.16	442.22	588.42		–	–	–	–	–	–
Left BA EM (mmHg)	NP	1,158.63	93.00	973.14	1,344.11	*P*	0.375	0.317	0.232	0.450	0.373	0.414
	HP	1,238.58	231.94	775.98	1,701.17	Boot. *P*	0.360	0.269	0.122	0.439	0.332	0.347
	GH	1,282.95	241.44	801.42	1,764.48		–	–	–	–	–	–
	PE	1,355.46	247.64	861.55	1,849.36		–	–	–	–	–	–

*MV, mean value; SE, standard error; 95% CI, 95% confidence interval; LL, Lower limit; UL, upper limit; Boot, bootstrapping; NP, non-pregnant women (n = 401); HP, healthy pregnant women (n = 10); GH, gestational hypertension (n = 8); PE, pre-eclampsia (n = 8); CCA, common carotid artery; Brachial artery. DD, end-diastolic diameter; cfPWV, crPWV, carotid-to-femoral and carotid-to-radial pulse wave velocity; PWV, pulse wave velocity; EM, elastic modulus*.

While PE showed elevated CCA EM *vs*. other groups (even comparing with GH), GH did not show significant differences in this parameter when compared to NP or HP (Table 3). In other words, this pregnancy status did not show the characteristic PE-associated increase in CCA stiffness or the HP-associated reduction in CCA stiffness. At the level of the BA, even though PE showed a trend toward higher BA stiffness, this finding did not reach statistical significance when compared with the other study groups (Table 3). Finally, CCA EM/BA EM, which was reduced in HP compared with NP, was similar between GH and PE vs. NP, although PE presented higher numerical values.

## Discussion

The present study is, to our knowledge, the first one to determine and compare, in a group of healthy non-pregnant and pregnant women with and without hypertensive disorders of pregnancy, the arterial SG by using different but complementary approaches considering central, peripheral, regional, and local arterial parameters. The main contributions of this study are:

First, when compared with NP status, HP was associated with unchanged PWV ratio but with a reduction in CCA EM/BA EM, in the setting of a generalized decrease in AS.

Second, when compared with NP and HP, PAH was associated with an “exaggerated rise” in the PWV ratio without any change in CCA EM/BA EM, in the setting of a blunt reduction in cfPWV and exaggerated crPWV reduction.

Third, the attenuation or even reversal of the central-to-peripheral SG observed in PAH was mainly driven by the changes in arterial stiffness observed in those women with PE.

### Healthy Pregnancy

While changes in central arteries have been largely described in different studies, changes in the peripheral AS have been inconclusive. Resting crPWV, which assesses mainly the upper limb AS (i.e., mostly muscular arteries), was found to be reduced in uncomplicated pregnant women in some (19, 33, 34) but not in all studies (20, 35, 36). Pregnancy-related changes in peripheral AS may also change over the course of the pregnancy (34).

In the present cross-sectional study, we found that both cfPWV and crPWV in HP were lower than those of NP in the third trimester. In addition, HP was also characterized by an increase in arterial diameters and a trend of showing lower local stiffness values (mainly CCA EM), all of which can explain the reduced regional AS. Despite these observations, the PWV ratio remained largely unchanged compared to NP (assessed by bootstrapping) because the relative reduction in both parameters seems to be counterbalanced. However, CCA EM/BA EM did show a significant reduction compared to NP. As previously mentioned, it has been demonstrated that the SG in normal non-pregnant populations works as a filter of excessive pressure energy transmission to certain microcirculatory beds (22). Thus, from a physiologic standpoint, the increased dampening function of the maternal aorta and the preservation of the SG would both have enhanced protective effects on the distal microcirculation limiting barotrauma and excessive shear forces, which would occur in an otherwise not adapted cardiovascular system to increase blood flow regimen (5, 33).

### Pregnancy-Associated Hypertension

On the other hand, in comparison with HP, both subgroups of PAH showed, although with different magnitudes, greater increments in both arterial diameters and CCA EM (BA EM was unchanged), along with a lower/blunted reduction in cfPWV and a higher elevation in PWV ratio. The higher cfPWV of PAH compared to HP suggests that the pregnancy-induced healthy decrease in the aortic stiffness did not occur in this group of patients (“impaired de-stiffening effect”). Of note, cfPWV in PAH was still significantly reduced compared to NP, a finding that was likely determined by an equilibrium between (i) larger diameters of central arteries (e.g., CCA) mainly present in women with PE (i.e., the larger arterial dimensions, the lower PWV; explained by Moens–Korteweg equation) and (ii) higher local AS found in both CCA [the higher EM, the higher PWV (Moens–Korteweg equation)] (Figure 1). The overall augmentation trend of arterial diameters in PAH could be an arterial compensatory response and/or BP dependent, and possibly, could be the reason behind the significant drop in crPWV levels when comparing PAH and NP. All these findings together determine that in PAH (but mostly in PE women) PWV and EM ratios become elevated (being in the latter case in the limit of significance). Regardless, the observed reduction in CCA EM/BA EM in HP compared to NP was not found in women with PAH compared to HP. Of note, when performing subgroup analysis in the group of women with PAH (Table 3), the blunt reduction in cfPWV values, the reduction in crPWV and the center-to-periphery arterial SG attenuation and/or reversal observed in the PAH group were mainly determined by women with PE. Strikingly, PE showed similar cfPWV values compared to NP, a detrimental finding in the setting of pregnancy, in where there is an expected 30 and 50% physiologic increase in SV and CO, respectively, to meet the increased metabolic needs of the developing fetus (37). This suggests that the aorta would not distend properly in the setting of the increase in effective circulating volume, or it would do it but at the expense of elevated aoBP. Moreover, a relative stiff aorta would provoke reflected pressure waves to travel faster from the periphery to the ascending aorta during the late systolic phase imposing inappropriate loads to LV. In non-pregnant populations, the increased aoBP, aortic stiffness, and pulsatile forward pressure wave in hypertensive patients have been shown to be associated with increased renal blood flow pulsatility, thereby explaining the association between PP, microalbuminuria, and renal microvascular damage (37). Similarly, Mitchell et al. reported that increased aortic stiffness and reduced wave reflection at the carotid-aorta interface were associated with excessive flow pulsatility, microvascular structural brain damage, and lower scores in various cognitive domains (14). Furthermore, the PWV ratio has shown independent clinical predictive value in different pathophysiologic circumstances, having this parameter higher prognostic value than cfPWV itself (11–13, 22).

The arteriolar network is a major site of resistance and reflections and the ultimate microcirculation protection against barotrauma and excessive shear forces. The loss of the SG has been associated with endothelial dysfunction, vascular myogenic response, and impaired organ perfusion (12). Thus, if the maternal arterial system suffers a loss or reversal of arterial SG (aortic PWV > peripheral PWV), pulsatile pressure could be either filtered by an increased arteriolar myogenic response but at the expense of reducing the blood flow or would not be sufficiently dampened and filtered damaging the distal microcirculation. Given the fetal metabolic needs, the placenta must operate at very high flow/low vascular resistance being second only to the kidney regarding blood flow rates per unit of tissue mass (8). Other low-resistance vascular beds, such as renal, hepatic, and cerebral circulation are also at risk of excessive pulsatility since microvascular pressure is also directly coupled with aoBP fluctuations (8) (Figure 1). Hence, the transmission of a higher pulsatile pressure into the placental and other low-resistance microcirculations might be highly likely in the setting of attenuation or reversal of SG. Taken together, the loss and/or reversal of arterial SG throughout the arterial tree could have a major role in the pathophysiology and clinical manifestations of PE, leading to secondary placental dysfunction (e.g., intrauterine growth restriction), renal (e.g., proteinuria) and hepatic damage (e.g., elevated liver enzymes, hematoma), and in other severe cases, cerebral dysfunction (5).

### Clinical and Physiological Relevance

The maternal arterial system during normal pregnancy, characterized by high metabolic needs and a high-flow state, requires not only a low peripheral vascular resistance but also an overall reduction in AS (central and peripheral) with preservation of the SG. This arterial adaptation would likely play a major role in facilitating adequate damping and filtering of excessive forward and reflected waves, optimization of BP, LV static and dynamic afterload, and microvascular network perfusion. Conversely, in PAH, and particularly PE, aortic stiffness does not decrease as physiologically required, and along with possible detrimental maladaptation of the peripheral arteries, dissipating the normal expected SG.

The loss or reversal of the SG, demonstrated to be deleterious in non-pregnant populations, would likely jeopardize the microcirculation in women with PAH, potentially leading to increased vascular myogenic response, endothelial dysfunction, and impaired organ perfusion. This arterial maladaptation syndrome observed in women with PAH could ultimately explain that obligate high-flow organs in the maternal circulation such as the kidneys, brain, and placenta are more susceptible to the adverse effects of the loss of SG having a potential role in the pathophysiology and clinical manifestations of PE.

### Strengths and Limitations

Our results should be analyzed in the context of strengths and limitations. To the best of our knowledge, there are no studies in the literature that have evaluated the SG in pregnant women complicated with PAH. Another important strength of this study is the robustness of the methodology employed to assess AS and its SG. We perform a comprehensive evaluation of AS (such as analysis of regional and local parameters) by using simple, non-invasive, robust, and reproducible methods. The use of applanation tonometry has been largely validated and PWV is regarded as the gold standard method for measuring regional AS. Regarding local stiffness and its gradient, we used a combination of high-resolution ultrasound and BP. In the latter case, in this study, aortic, and brachial BP was used to quantify central and peripheral AS levels, respectively. This should be considered as a strength since previous studies have quantified CCA stiffness by using brachial PP, which could lead to inaccuracies of the parameters.

This study has certain limitations. First, the sample size of our group of pregnant women is relatively small. To overcome this limitation, we used bootstrapping, a statistical method that creates a new sample of observations of the variables by randomized re-sampling with replacement based on the original observations. Although this method has its own advantages and disadvantages, even in this context, the biggest mistake we can make is not to generate a type I error (finding differences when in fact there are no differences), but to generate a type 2 error (not finding differences when in fact there are differences). Consequently, we have been “conservative,” in the fact that we may miss significant differences in cases where potentially there are. Second, since this is a cross-sectional study, it provides no data on longitudinal pregnancy-related temporal variations in variables of interest. Prospective studies are clinically needed to assess whether women showing a reversal of PWV ratio or absence of CCA EM/BA EM reduction are at risk of developing obstetric complications. Third, in this work, the concept of SG was presented as “static or unchanged,” rather than the composite of (i) “fixed or stable” [e.g., age-dependent vascular (intrinsic) stiffness level] and (ii) “variable or adjustable” (e.g., endothelial and vascular smooth muscle ability to temporally adjust the AS levels) (38). The systematization of recording conditions is necessary to evaluate AS-related parameters considering the existence of modulating factors. In this work, to systematize the measurement and to minimize the impact of sources of variability, AS levels were determined at rest, under stable hemodynamic conditions.

## Conclusions

Compared with NP status, HP was associated with unchanged PWV ratio but with a reduction in CCA EM/BA EM, in the setting of a generalized decrease in AS.

Compared with NP and HP, PAH was associated with an “exaggerated rise” in the PWV ratio without any change in CCA EM/BA EM, in the setting of a blunt reduction in cfPWV and exaggerated crPWV reduction.

The attenuation or even reversal of the SG observed in PAH was mainly driven by the changes in arterial stiffness observed in those women with PE.

## Data Availability Statement

The original contributions presented in the study are included in the article/Supplementary Material, further inquiries can be directed to the corresponding author/s.

## Ethics Statement

The studies involving human participants were reviewed and approved by Centro Hospitalario Pereira-Rossell Ethic Committee and Hospital de Clínicas. The patients/participants provided their written informed consent to participate in this study.

## Author Contributions

MP, JT, DB, and YZ contributed to the conception and design of the study. JT, YZ, and DB performed the cardiovascular non-invasive recordings and constructed and organized the database. YZ and DB performed the statistical analysis. MP, JT, DB, and YZ wrote the first draft of the manuscript. CS and AD performed revisions and critically discussed the complete manuscript. All authors, read, and approved the submitted version.

## Funding

This research was funded by the Agencia Nacional de Investigación e Innovación (ANII), Grant No/code. PRSCT-008-020 and extra-budgetary funds provided by the CUiiDARTE Center (DB, YZ).

## Conflict of Interest

The authors declare that the research was conducted in the absence of any commercial or financial relationships that could be construed as a potential conflict of interest.

## Publisher's Note

All claims expressed in this article are solely those of the authors and do not necessarily represent those of their affiliated organizations, or those of the publisher, the editors and the reviewers. Any product that may be evaluated in this article, or claim that may be made by its manufacturer, is not guaranteed or endorsed by the publisher.

## Abbreviations

aoBP: Central aortic blood pressure
AS: Arterial stiffness
BA: Brachial artery
baBP: Brachial artery blood pressure
baDBP: Brachial artery diastolic blood pressure
baSBP: Brachial artery systolic blood pressure
BP: Blood pressure
CCA: Common carotid artery
cfPWV: Carotid-to-femoral pulse wave velocity
CO: Cardiac output
CRFs: Cardiovascular risk factors
crPWV: Carotid-to-radial pulse wave velocity
DD: End-diastolic arterial diameter
EM: Pressure-strain elastic modulus
GH: Gestational hypertension
HP: Healthy pregnancy
HR: Heart rate
IMT: Intima-media thickness
LL: Lower limit
MBP: Mean blood pressure
MV: Mean value
PAH: Pregnancy-associated hypertension
PE: Preeclampsia
PP: Pulse pressure
PWA: Pulse wave analysis
PWV: Pulse wave velocity
SBP: Systolic Blood Pressure
SD: Standard deviation
SG: Stiffness gradient
SV: Stroke volume
SysD: Peak systolic arterial diameter
UL: Upper limit.
